# Prognostic impact of circulating Her-2-reactive T-cells producing pro- and/or anti-inflammatory cytokines in elderly breast cancer patients

**DOI:** 10.1186/s40425-015-0090-0

**Published:** 2015-10-20

**Authors:** Jithendra Kini Bailur, Evelyna Derhovanessian, Brigitte Gueckel, Graham Pawelec

**Affiliations:** Department of Internal Medicine II, Centre for Medical Research, University of Tuebingen, Waldhoernlestr 22, 72072 Tuebingen, Germany; Radiology Clinic, Diagnostic and Interventional Radiology, University Hospital Tuebingen, Tuebingen, Germany; Present Address: BioNTech AG, Mainz, Germany; School of Science and Technology, College of Arts and Science, Nottingham Trent University, Nottingham, UK; Present Address: Yale Cancer Center, Yale University School of Medicine, New Haven, CT USA

**Keywords:** T-cells, Breast cancer, Her-2, Elderly, Tumour-associated antigens, Cytokines, Immunosenescence

## Abstract

**Background:**

Treating elderly breast cancer patients remains a challenge but the increasing availability of immunotherapeutic approaches instills optimism that these tumours may also be susceptible to immune control. Because aging leads to a number of alterations in the immune system (“immunosenescence”) reflecting potential exhaustion which could compromise immunomodulatory antibody therapy, here we have assessed the immunocompetence of elderly breast cancer patients compared with a group of younger patients, and related this to the 5-year survival of the former.

**Methods:**

T-cell responses to Her-2 peptide pools *in vitro* were assessed by analyzing pro- and anti-inflammatory cytokine production by CD4+ and CD8+ T-cells in 40 elderly and 35 younger breast cancer patients.

**Results:**

The proportions of older and younger patients whose peripheral T-cells responded to Her-2 peptides *in vitro* were found to be similar, although a significantly higher fraction of younger patients possessed IL-2-producing CD4+ Her-2-reactive T-cells than in the elderly (*p* = 0.03). However, IL-2 production did not impart a survival benefit to the latter. In contrast, there was a survival benefit of possessing Her-2-reactive CD8+ T-cells, but this was abrogated in patients if they also had CD4+ Her-2-responsive T-cells that producedIL-5 and/or IL-17 (*p* = 0.01). This resulted in a 5-yr survival rate of only 29 % compared to 76 % for patients whose her-2-reactive CD4+ T-cells did not produceIL-5 and/or IL-17. Additionally, patients whose CD8+ T-cells produced TNF had a significantly better survival than those that did not (93 % compared to 52 %, *p* = 0.01), whereas no survival benefit was attributable to possessing IFN-γ-producing cells.

**Conclusions:**

Elderly breast cancer patients appear perfectly immunocompetent to respond to Her-2 peptide pools *in vitro*, with response patterns very similar to younger patients. The nature of this response is associated with 5-year survival of these elderly patients, suggesting that boosting anti-tumor responses and modulating the nature of the T-cell response is likely to be effective even in potentially immunosenescent elderly breast cancer patients, and might be useful for predicting which patients are most likely to benefit from such treatments.

**Electronic supplementary material:**

The online version of this article (doi:10.1186/s40425-015-0090-0) contains supplementary material, which is available to authorized users.

## Background

The prevalence of most types of cancer increases with age. One reason for this may be that aging is associated with dysregulated immunity, which could contribute to increased susceptibility to infections and cancer [1]. Treating elderly breast cancer patients remains a major challenge. The elderly are underrepresented in clinical trials, of which there are hardly any that have specifically considered elderly cohorts. Generally, the same treatment regimens are applied to both younger and older cancer patients, but the latter are not able to tolerate chemotherapy or radiotherapy to the same extent and may thus be under-treated [2, 3]. Although there are many suggestions as to how to treat elderly breast cancer patients [4-6], and considering the recent breakthroughs in immunotherapy of melanoma and other cancer types, more investigations need to be conducted to understand the immune system of these patients and the potential role of ageing and immunosenescence in treatment outcome. The hallmarks of immunosenescence may be observed chronologically earlier in chronic viral infections and cancer [7, 8]. There is a number of alterations that are known to occur with aging [9, 10], which include changes in the number and distribution of T and B lymphocytes and their differentiation states [8, 11]. As T-cells play a crucial role in adaptive immunity, their efficient activation to defend against cancer or viral infection is important. Dendritic cells (DCs) are necessary for activating the T-cells efficiently. Aging may also result in alterations of important co-receptors on DCs that could result in weakened T-cell response, possibly reducing anti-cancer immunity [12, 13]. Generally, higher levels of serum inflammatory markers are also observed in the elderly, which could play a role in the activation of immunosuppressive cell subsets making it more difficult to resist cancer [14, 15]. Thus, there are several loci at which immunosenescence could interfere with anti-cancer immunity and accordingly compromise immunotherapies. The recent striking clinical success of employing immunomodulatory antibodies such as anti-CTLA-4, PD-1 or PDL-1which “take the brakes off” immunity and are showing extremely promising results in some cancers in a proportion of treated patients makes it even more important to determine the effect of age on the immune status of cancer patients [16-18]. Such therapies are likely to be effective only when the patient remains capable of mounting an anti-cancer immune response. In elderly patients, immunosenescence could compromise these responses.

Earlier, we showed that the presence of peripheral T-cells responding to certain tumor-associated antigens (TAA) in stage IV melanoma was associated with longer survival and that the pro- or anti-inflammatory nature of the T cell response influenced the strength of this association [19, 20]. Thus, T-cell responsiveness to TAA is an important prognostic biomarker of survival. Melanoma patients tend to be relatively young compared to most other solid cancer patients, and so we asked whether this type of biomarker reflecting T-cell functional integrity would also be informative in older patients. We therefore elected to examine this issue in a cohort of some of the oldest such patients, namely in elderly female breast cancer patients. We had previously reported that elderly patients with *in vitro* CD8+ T-cell responses against pooled Her-2 peptides survived longer than those who did not [21], suggesting that immunosenescence had not compromised responsiveness and that immunomodulatory therapies should also be effective in these patients. Here, we compared the immunocompetence of these elderly patients with a group of younger patients and found that they were indeed similar in this respect. We have continued these first studies on the elderly to dissect the nature of their CD4+ and CD8+ T-cell responses to Her-2 peptides in relation to their overall survival, where we were able to show the association of certain pro- and anti-inflammatory cytokines produced by CD4+ and CD8+ T-cells with overall survival, analogous to similar findings in melanoma [20].

## Results

### T-cell responses to Her-2

A majority of the elderly(97 %, *n* = 38) and all younger (100 %, *n* = 35) breast cancer patients analyzed had *in vitro* T cell responses to mixtures of Her-2 peptides. FACS plots from a representative donor are shown in Additional file 1: Table S2. CD4+ T-cell responses to Her-2 were observed in most individuals in the case of both older (32/38, 87 %) and younger (33/35, 94 %) patients, whereas only 18 of the former (47 %) and 21 of the latter (60 %) possessed CD8+ T-cells responding to Her-2 peptides. ThisCD8+ T-cell response was present irrespective of whether the patients also had a CD4+ T-cell response to Her-2. Taking advantage of our ability to analyze 6 different cytokines simultaneously by intra-cellular staining of individual T-cells by flow cytometry, we grouped the Her-2 responders according to the cytokines produced by their CD4+ and CD8+ T-cells.

### CD8+ T-cell responses to Her-2

As described above, a CD8+ T-cell response to Her-2, defined as the production of any one of the 6 tested cytokines, was observed in 18/38 (47 %) older and 21/35 (60 %) younger patients. In a high proportion of these patients, CD8+ T-cells responding to Her-2produced the pro-inflammatory cytokines TNF (14/18, 78 % in old; 16/21, 76 % in young) and IFN-γ (13/18, 72 % in old; 18/21, 86 % in young).Only a small proportion of CD8+ T-cells produced IL-2 and IL-10 in either old or young patients (Tables 1 and 2).

**Table 1:** Cytokines produced by Her-2 responders in old breast cancer patients

Old (*n* = 38)					
CD4	Response	No. of patients	% Dead	5-year survival rate	*P* value
TNF	Yes	27	37	60	0.1
	No	11	18	81	
IFN-γ	Yes	23	43	57	0.1
	No	15	13	84	
IL-5	Yes	7	57	43	0.1
	No	31	23	73	
IL-10	Yes	3	33	67	0.8
	No	35	31	65	
IL-2	Yes	13	46	54	0.3
	No	25	24	74	
IL-17	Yes	3	67	33	0.3
	No	35	29	70	
IL-5 and or IL-17	Yes	10	50	29	0.01
	No	28	25	76	
CD8					
TNF	Yes	14	7	93	0.01
	No	24	46	52	
IFN- γ	Yes	13	23	76	0.3
	No	25	36	62	
IL-10	Yes	2	50	50	0.7
	No	36	31	68	
IL-2	Yes	1	0	100	0.5
	No	37	32	66	

Results of survival analysis according to Kaplan Meier method and *p* values from Mantel-Cox (log-rank) test

**Table 2:** Cytokines produced by Her-2 responders in young breast cancer patients

Young (*n* = 35)				
CD4	Response	No. of patients	% Dead	*P* value
TNF	Yes	27	7	0.5
	No	8	0	
IFN-γ	Yes	28	7	0.6
	No	7	0	
IL-5	Yes	8	25	0.04
	No	27	0	
IL-10	Yes	4	0	0.7
	No	31	6	
IL-2	Yes	22	9	0.4
	No	13	0	
IL-17	Yes	6	17	0.6
	No	29	3	
IL-5 and or IL-17	Yes	12	17	0.1
	No	23	0	
CD8				
TNF	Yes	16	13	0.3
	No	19	0	
IFN-γ	Yes	18	11	0.3
	No	17	0	
IL-10	Yes	2	0	0.3
	No	33	6	
IL-2	Yes	2	0	0.8
	No	33	6	

Results of survival analysis according to Kaplan Meier method and *p* values from Mantel-Cox (log-rank) test

### CD4+ T-cell responses to Her-2

Analyzing the nature of the CD4+ T-cell responses to Her-2 peptides, we observed that these cells mainly produced the pro-inflammatory cytokines TNF (27/32, 84 % in old; 27/33, 82 % in young), IFN-γ (23/32, 72 % in old; 28/33, 85 % in young) and, unlike for CD8+ T-cells, also IL-2 (13/32, 41 % in old; 22/33, 67 % in young). The higher fraction of younger relative to older patients whose CD4+ T-cells produced IL-2 in response to Her-2 was statistically significantly different (*p* = 0.03). As for CD8+ T-cell responses, IL10, IL-5 and IL-17 were rarely produced by either young or old patients (Tables 1 and 2).

### The T-cell response to Her-2 correlates with overall survival

**Fig. 1:**
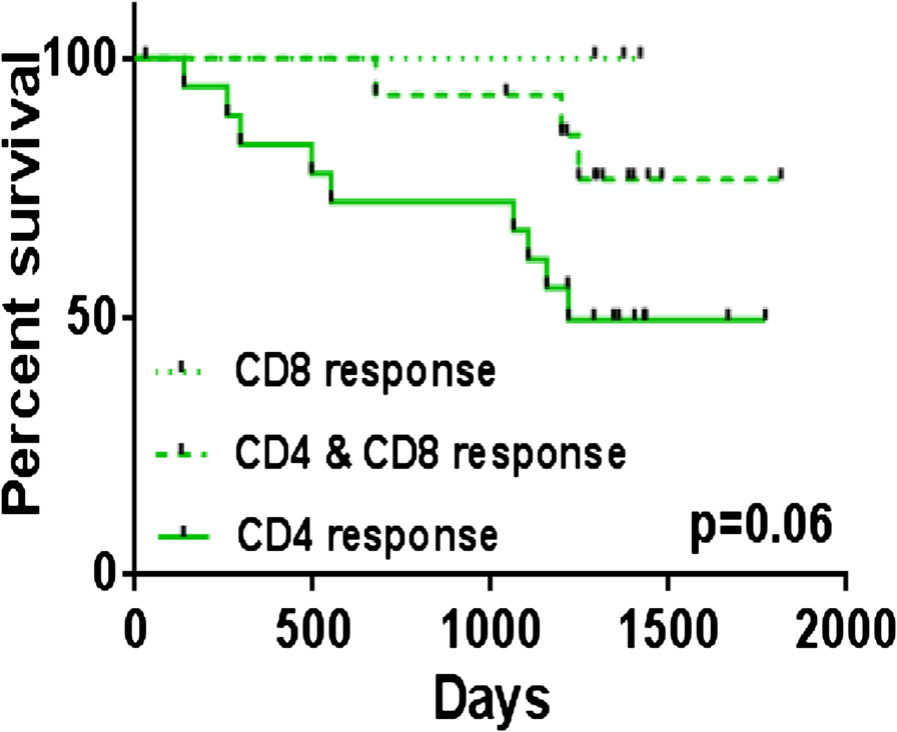
Survival analysis of elderly breast cancer patients according to CD8+ T-cell responses to Her-2peptides *in vitro*. Survival analysis of elderly patients (*n* = 38) grouped according to CD8+ T-cell responses to Her-2 (CD8 response), CD4+ and CD8+ T-cell responses (CD4 & CD8response) and CD4+ T-cell responses to Her-2 (CD4 response)

Previously, we had shown that elderly patients who had a CD8+ T-cell response to Her-2 had a survival advantage over those who did not(*p* = 0.03). The overall (5-year) survival rate was 52 % for patients without a CD8+ T-cell response to Her-2, compared with 82 % for those had a CD8+ T-cell response. There was only 17 % mortality in the group of patients with a CD8+ T-cell response to Her-2 whereas this was 45 % for patients without a CD8+ T-cell response. In the present study, we stratified patients according to whether they had only a CD8+ T-cell response, only a CD4+ T-cell response or both CD4+ and CD8+ T-cell responses to Her-2 peptides, and performed a Kaplan-Meier analysis (Table 3). We confirmed a survival benefit for patients with Her-2-reactive CD8+ T-cells as observed in our earlier study, but there was no survival advantage for patients with a CD4+ T-cell response to Her-2. Moreover, those patients with a CD8+ T-cell response to Her-2 who also had a CD4+ T-cell response had poorer survival than those with only a CD8+ T-cell response. A significant early negative impact on survival can also be observed for patients with no CD8+ T-cell response to Her-2 (Gehan Breslow test: *p* = 0.02) (Fig. 1). The 5-year survival rate for patients with a solely CD4+ T-cell response was 49 % compared to 77 % and 100 % for those with combined CD4+ and CD8+ T-cell responses or only a CD8+ T-cell response, respectively. Survival analyses were not possible in the younger patients because only two had died. Nonetheless, these two had both CD4+ and CD8+ T-cell responses to Her-2.

**Table 3:** Phenotype of Her-2 responders in older patients

Response to Her-2		No. of patients	Percentage dead	*p* value
CD4 response	Yes	33	36 %	0.1
	No	4	0 %	
CD8 response	Yes	18	13 %	0.03
	No	19	47 %	
CD4 and CD8 response	Yes	14	21 %	0.2
	No	23	39 %	

Results of survival analysis according to Kaplan Meier method and p values from Mantel-Cox (log-rank) test

### Impact of the presence of CD4+ T-cells producing IL-5 and IL-17 on survival

**Fig. 2:**
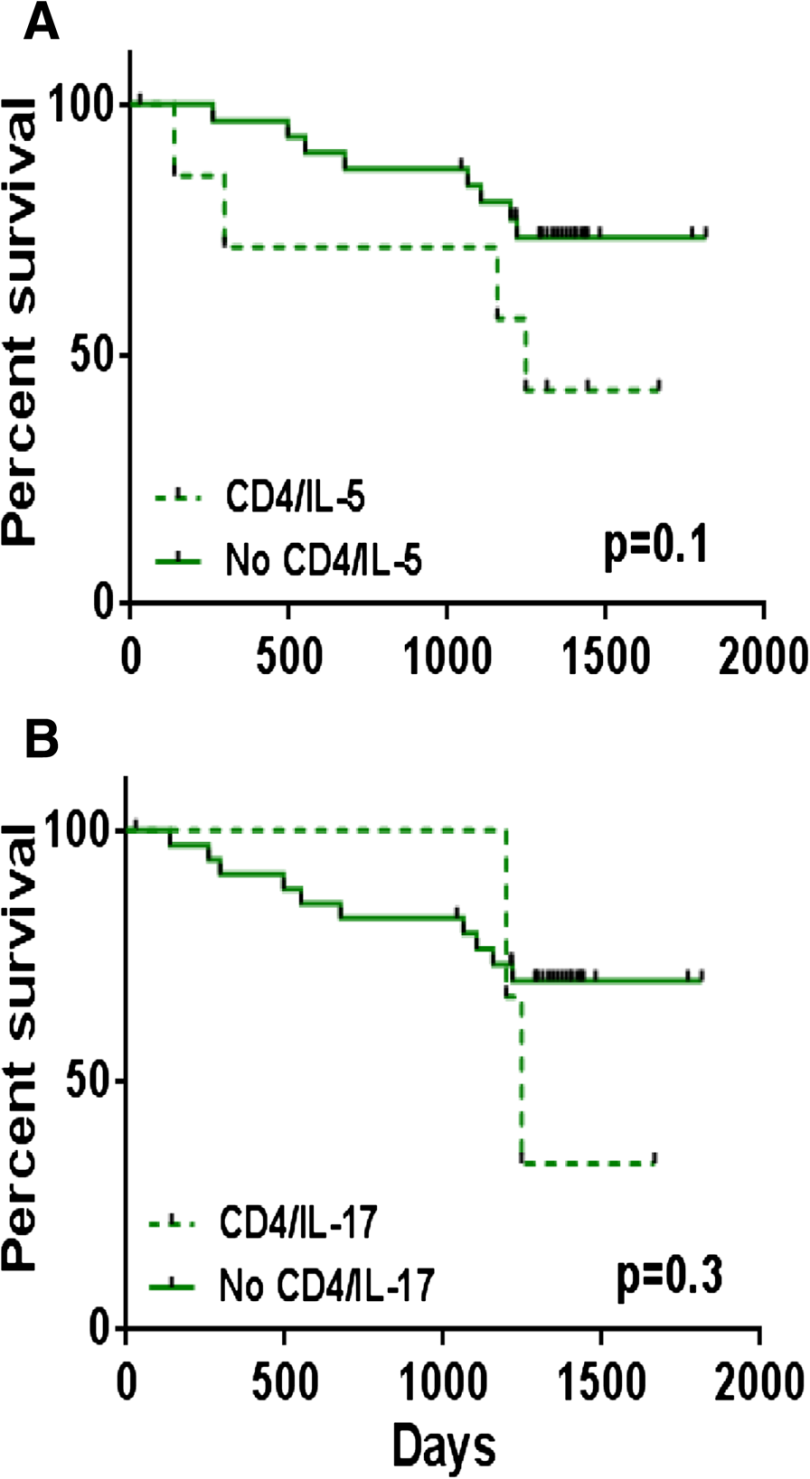
Survival analysis of elderly breast cancer patients according to IL-5 or IL-17 production by Her-2-stimulated CD4+ T-cells. **a** Patients grouped according to IL-5 production; **b** IL-17 production

**Fig. 3:**
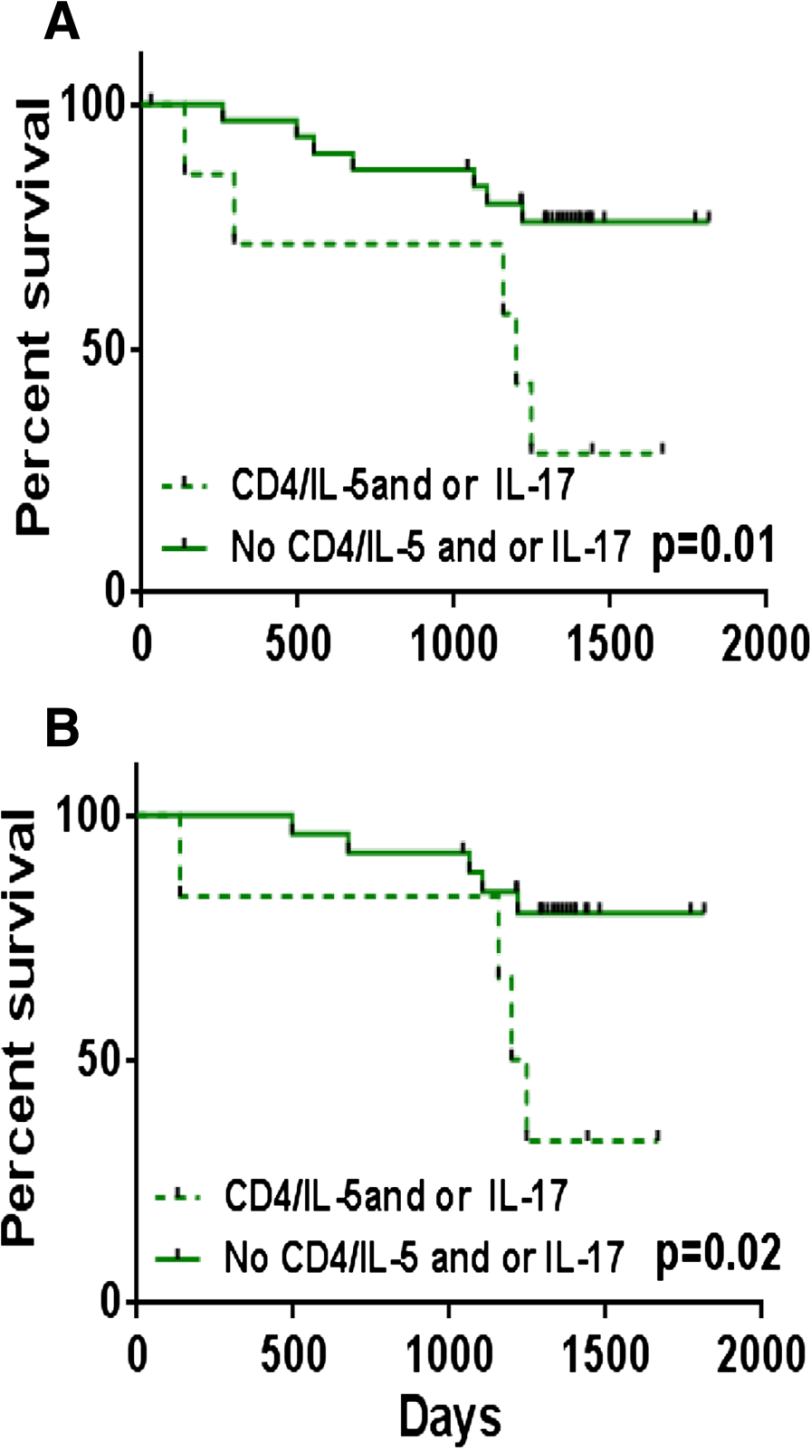
Survival analysis of elderly breast cancer patients according to IL-5 and/or IL-17 production by Her-2-stimulated CD4+ T-cells. **a** patients grouped according to IL-5 and/or IL-17 production in all patients and **b** in non-metastatic patients only

Patients were stratified according to the cytokines produced by their CD4+ or CD8+ T-cells. Considering the elderly breast cancer patients, Kaplan-Meier survival analysis showed that patients whose CD4+ T-cells produced IL-5 tended to have poorer outcomes (*p* = 0.1, Fig. 2a) with a survival rate of 43 % compared to 73 % for patients whose CD4+ T-cells did not produce IL-5 when stimulated with Her-2 peptides. A weaker trend towards a similar impact on survival was observed for patients whose CD4+ T-cells produced IL-17 (*p* = 0.3, Fig. 2b). Here, those whose CD4+ T-cells produced IL-17 had a 33 % 5-year survival compared to 70 % for those who did not produce IL-17. Despite the lack of statistical significance when considering IL 5 and IL 17 production separately, when we analyzed patients whose CD4+ T-cells produced IL-5 and/or IL-17, we did observe a significant impact on the overall survival (*p* = 0.01, Fig. 3a) in these elderly breast cancer patients. Thus, the survival rate for patients whose CD4+ T-cells produced IL-5 and/or IL-17 was 29 % compared to 76 % for the patients without these cytokines. From the Kaplan-Meier analysis of different parameters in the elderly we observed the expected impact of metastasis on survival (Additional file 2: Table S1). Limiting the analysis to non-metastatic elderly breast cancer patients only, we still observed a similar negative impact on survival for the patients whose CD4+ T-cells produced IL-5 and/or IL-17 (*p* = 0.02, Fig. 3b). Here, the survival rate was only 33 % compared to 80 % for patients whose CD4+ T-cells did not produce these cytokines.

Analogous observations were also made anecdotally in the younger patients, where those whose CD4+ T-cells produced IL-5 had poor survival, with 25 % (2/8) of the IL-5-producing patients dying, compared to none whose CD4+ T-cells did not produce IL-5.

### Impact of TNF-producing CD8+ T-cells on survival

**Fig. 4:**
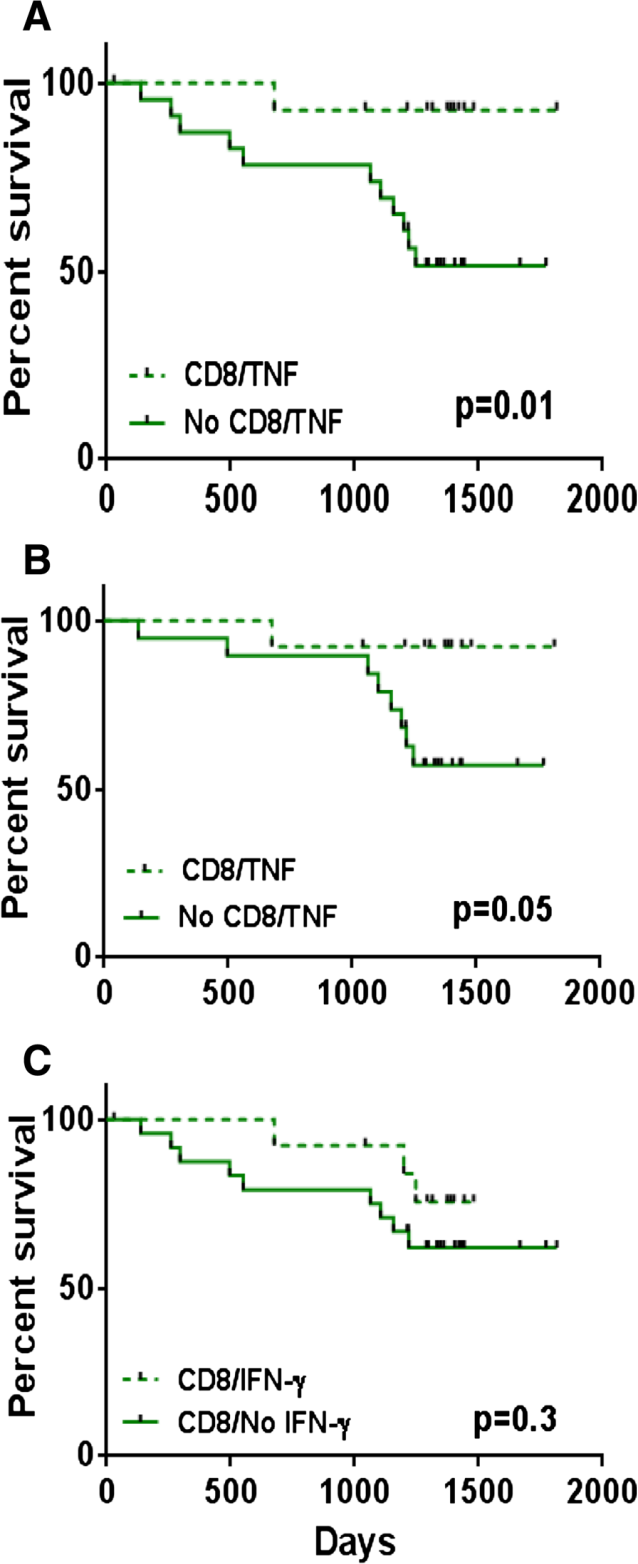
Survival analysis of elderly breast cancer patients according to CD8+ T-cell responses to Her-2 peptides in vitro. **a** patients grouped according to TNF production in all patients and **b** in non-metastatic patients only; **c** patients grouped according to IFN-γ production (all patients)

We previously reported the beneficial effects of having a CD8+ T-cell response to Her-2. Here, we investigated the function of these Her-2 specific CD8+ T-cells to identify the factors associating with survival benefit in this cohort. We observed that patients whose CD8+ T-cell produced TNF had a significantly better survival than those whose CD8+ T-cells did not produce TNF, when all elderly patients were included in the analysis (*p* = 0.01, Fig. 4a). The survival rate for the patients whose CD8+ T-cells produced TNF was 93 % compared to 52 %, for those with no TNF-producing CD8+ T-cells. This survival advantage was still observed when only non-metastatic elderly patients were considered (*p* = 0.05, Fig. 4b). In the case of non-metastatic patients, these figures were 92 % and 57 %, respectively. No survival associations could be observed in young.

### Impact of IFN-γ producing CD8+ T-cell on survival

Here, we grouped the patients according to CD8+ T-cell production of IFN-γ. Unlike the findings with TNF above, unexpectedly, Kaplan-Meier survival analysis indicated no significant differences between IFN-producers and non-producers both in young and old (*p* = 0.3, Fig. 4c).

## Discussion

In the present study, we investigated associations of the presence of circulating Her-2-reactive CD4+ and CD8+ T-cells with survival in older breast cancer patients relative to their pro- and anti-inflammatory cytokine production on stimulation, and compared the immunocompetence of the elderly, as assessed in this manner, with that of younger patients. Previously, we had reported a survival benefit accruing to patients having a CD8+ T-cell response to Her-2 [21]. To test the hypothesis that the immune system in the elderly may be compromised due to aging, here we investigated differences in T-cell responses to Her-2 in younger and older breast cancer patients. To the best of our knowledge, this is the first study to investigate the phenotype and function of Her-2 reactive T-cells, or those reactive to any tumor-associated antigen, in young and old breast cancer patients.

In the present study, stratifying the patients according to those having a CD8+ T-cell response alone (positive impact on survival as previously shown), or both CD4+ and CD8+ T-cell responses, or only a CD4+ T-cell response revealed a trend towards a negative general survival impact for CD4+ T-cell reactivity in older patients. These results clearly confirmed the importance of having a CD8+ T-cell response to Her-2 but also showed that there was reduced survival when a CD4+ T-cell response to Her-2 was also present in the same patient. This implies that at least in terms of responses to Her-2, CD4+ T-cell reactivity to this TAA is likely to have unanticipated negative effects. CD4+ regulatory T cells would be the obvious candidates for the cells mediating these negative effects. The lack of CD8+ T-cell responses in patients with poorer survival could also be due to the presence of increased levels of Tregs. This would be consistent with other reported studies showing that Tregs could control the T-cell response against certain TAAs in an antigen-specific manner [22, 23]. We were not able to test for classic Treg phenotypes in the current study due to the limitations of the flow cytometric technique, but will focus on this question in future studies. We can only say at this juncture that one cytokine commonly implicated in Treg function, IL-10, does not seem to be responsible for these results, whereas two other cytokines not generally implicated in Treg function, namely IL-5 and IL-17, do seem to play important roles. Thus, functional analysis of 6 different cytokines allowed us to group patients according to the cytokines produced by the CD4+ and CD8+ T-cells responding to Her-2. Survival analysis showed that the presence of CD4+ T-cells producing IL-5 and/or IL-17 had a negative impact on survival in the elderly (*p* = 0.01). Compared to other anti-inflammatory cytokines, the role of IL-5 in peripheral cells seems to be under-investigated. Studies have indicated that IL-5 may facilitate lung metastasis, and high expression of IL-5 in the tumor was also negatively associated with survival [24, 25]. A potential role in immune surveillance has been proposed [26]. Similarly, there are few data on IL-17 in this context. Although IL-17 is primarily considered a pro-inflammatory cytokine, its role in cancer is still unclear. Some studies have shown anti-tumor effects of IL-17, so that it has been proposed for use as a cancer immunotherapeutic [27, 28]. However, tumor-infiltrating lymphocytes with a high level of IL-17-positivity correlate with poor prognosis in some studies [29, 24], and an association of IL-17 with Tregs in the tumor has been reported [29]. We also need to bear in mind that not only Tregs, but also myeloid-derived suppressor cells (MDSCs) may play a role in suppressing T-cell activity. We previously reported the possible suppressive role of MDSCs and Tregs in relation to T-cell responses to Her-2 and also the possible indirect role of these immunosuppressive subsets in breast cancer [21]. MDSCs are also associated with IL-17, as they are able to mediate IL-17 production by naïve T-cells, depending on the cytokines produced by the MDSCs themselves [30]. Consistent with the present report, our previous study investigating the role of IL-17 in the context of CD4+ T-cell responses to TAAs documented a negative impact ofIL-17-producing T-cells in late-stage melanoma [20]. There are several other cytokines such as IL-4 and IL-13 that are known to have a negative impact on survival [31, 32] and which would have been interesting to examine here, especially as we previously found that IL 4 was relevant in melanoma. IL-13 is known to regulate cancer invasion and metastasis in different cancers [31, 32]. In our previous study on melanoma, we observed a negative impact on survival for the patients who produced IL-4 reactive to Melan-A [20]. IL-4 is also known to trigger the cancer proliferation mechanism [33]. Not only these but also IL-6 and IL-1 have shown a negative impact on survival and tumor progression [34, 35]. However, the number of different variables that could be tested at the same time was limited and we were unable to include these other cytokines in the present analysis.

The role of the pro-inflammatory cytokines IL-2, IFN-γ and TNF in cancer is usually considered to be well-established. They play an important role in inducing a protective immune response [36] and IL-2 has long been used as an immunotherapeutic drug which can result in a small minority of very durable responses [37]. In the present study, one of the few differences that we observed between younger and older patients was that significantly lower proportion of the latter possessed Her-2-reactive IL-2 producing CD4+ T-cells, possibly contributing to better survival of the former. However, whether or not an elderly patient possessed IL-2-producing CD4+ T-cells had no impact on survival.

IFN-γ is known to play an important role in anti-tumor activity [38, 39] and has also been considered as an immunotherapeutic drug to treat cancer patients [40, 41]. In our study, however, we could discern no significant survival advantage for patients with Her-2-reactive CD8+ T-cells producing IFN-γ, although there was a survival advantage for patients who had Her-2-reactive TNF-producing CD8+ T-cells. TNF is involved in a number of important cellular functions including cell proliferation, survival and death and has been used in patients with locally advanced tumors [42]. In the present study, we observed that patients whose CD8+ T-cells produced TNF had a significant survival advantage over those who did not, indicating the importance of Her-2-reactive TNF-producing CD8+ T-cells for these elderly breast cancer patients.

## Conclusions

From our results, we propose that the nature of the anti-tumor T-cell response and the TAA targeted by T-cells present in the circulation is important and has an impact on patient survival. Considering the results presented in this study, depletion of Her-2-specific IL-5- and IL-17-producing CD4+ T-cells and enrichment of TNF-producing CD8+ T-cells for adoptive T-cell therapy would be important in breast cancer. Thus, considering our previous results, it is not only immunosuppressive cells like Tregs and MDSCs that might have to be targeted but it is also important to consider the specific cytokines produced by the CD4+ and CD8+ T-cell that are reactive to, in this case, Her-2. This study may also provide some mechanistic insights for a better understanding of the immune system in elderly and younger breast cancer patients. In any event, the findings that elderly breast cancer patients are not immunosenescent according to the assays conducted in this work augur well for the efficacy of immunomodulatory antibodies even in older patients.

## Methods

### Patients

The study included 40 elderly and 35 younger patients with breast cancer (Additional file 3: Figure S1). Blood samples from patients with different TNM stage were collected between March and November 2009, at the University Hospital Tuebingen. Blood was drawn prior to any treatment or surgery. Standard Ficoll-Hypaque gradient centrifugation was used to isolate the peripheral blood mononuclear cells (PBMCs) before they were cryopreserved for experimental purposes. The study was approved by the Institutional Ethics Committee of University Hospital Tuebingen (71/2009BO2) and a waiver of informed consent was granted for this study.

### Detection of tumour-associated antigen-reactiveT-cells

To assess T-cell reactivity to Her-2, a 12-day *in vitro* culture was performed as described previously [21]. First, after thawing carefully, washing extensively, and assessing viability, PBMCs (1x10^6^) were cultured in X-Vivo 15 defined medium (Lonza) supplemented with IL-4 (5 ng/ml: Sandoz, Basel, Switzerland) and IL-7 (5 ng/ml: Sterling-Winthrop, US), on day 0. On day 1, the PBMCs were stimulated with mixtures of Her-2 15-mer overlapping peptides (with an overlap of 11 amino acids) (PepMix, JPT Technologies, Berlin, Germany) at a concentration of 1 μg/ml. The cells were supplemented with IL-2 (40U/ml: Chiron Behring GmbH, Marburg, Germany) on day3. On day12, after harvesting and washing, the cultured T-cells were re-stimulated (0.4-0.5 x 10 ^6^ cells/well) with Her-2 PepMix at a concentration of 1 μg/ml or left unstimulated as a negative control for 12 hours. Golgi-plug (BD Biosciences) was added at 1 μl/ml to all cultures. Patients’ cells were stimulated with influenza nucleoprotein (NP) and membrane protein (M1) peptides as a positive control, as all subjects have been exposed to influenza during their lives and all possess T cells responsive to these peptides. After harvesting and washing, the cells were incubated with Gamunex (Talecris) to block Fc receptors, and with ethidium monoazide (EMA,MoBiTec GmbH, Goettingen, Germany), a marker for dead cells. Intracellular cytokine staining was performed after fixing and permeabilizing the cells with Cytofix/Cytoperm (BD Biosciences). Cells were simultaneously stained with CD3-PO (Invitrogen), CD4-Pacific Blue, TNF-FITC, IL-2-Alexa Fluor-700, IL-5-PE (Bio Legend), CD8-APC-Cy7, IFN-γ-PE-Cy7 (BD Biosciences), IL-10-APC (Miltenyi Biotech) and IL-17-PerCP-Cy5.5 (eBioscience). Following the staining, cells were measured on a BD-LSR-II flow cytometer using the FACS-Diva software (BD-Biosciences).

### Flow cytometry data analysis

Data were analyzed using FlowJo software (Tree Star Inc.) as shown earlier [21]. To detect cytokine-producing cells, the stimulated samples were compared with unstimulated (negative) control and the response was considered positive when at least one cytokine was produced by the stimulated sample (representative FACS plot shown in Additional file 1: Table S2), defined as an at least two-fold increase in the peptide-stimulated culture compared to the unstimulated negative control, as established in earlier studies [19, 21].

### Statistical analysis

Chi-square testing was performed to compare independent groups and Kaplan-Meier analysis was performed (Log-rank test and Gehan-Breslow test) for the survival estimates. Graph Pad Prism 6 was used to perform this analysis. A value of *p* < 0.05 was considered statistically significant.

## Additional files

Additional file 1: Table S2. Kaplan-Meier analysis of clinico-pathological parameters in older patients. (DOCX 17 kb) Additional file 2: Table S1. Clinico-pathological characteristics of younger and older patients. (DOCX 15 kb) Additional file 3: Figure S1. CD8+ and CD4+ T-cell response to Her-2: A representative plot of control and Her-2-stimulated cytokine (eg.TNF-producing CD8+ T-cells and CD4+ T-cells). (PPTX 89 kb)

## Abbreviations

Her-2: Human epidermal growth factor receptor-2
IL: Interleukin
CTLA-4: Cytotoxic T-lymphocyte associated protein-4
PD-1: Programmed cell death protein-1
PDL-1: Programmed death ligand-1
