# Supplementary material for: Prognostic impact of circulating Her-2-reactive T-cells producing pro- and/or anti-inflammatory cytokines in elderly breast cancer patients
Source: J Immunother Cancer. 2015 Oct 20;3:45. doi: 10.1186/s40425-015-0090-0 (PMC4617728; doi:10.1186/s40425-015-0090-0)
Supplement: Additional file 1: Table S2. — Kaplan-Meier analysis of clinico-pathological parameters in older patients. (DOCX 17 kb) [file 40425_2015_90_MOESM1_ESM.docx]

**Table S2: Kaplan-Meier analysis of 5-year survival according to clinico-pathological parameters in elderly patients**

|  |  |  | **Overall survival rate** | | |
| --- | --- | --- | --- | --- | --- |
| **Factor** | **N** | **% Dead (5years)** | **%** | **95% CI** | **P** |
| **Metastasis** |  |  |  |  | **0.03** |
| No | 33 | 28 | 72 | 0.01--0.8 |  |
| Yes | 6 | 50 | 40 | 1.1-65.6 |  |
| **Her-2 status** |  |  |  |  | 0.3 |
| Neg (low) | 34 | 26 | 72 | 0.05-2.8 |  |
| Pos | 4 | 50 | 50 | 0.3-17.9 |  |
| **Oestrogen receptor** |  |  |  |  | 0.3 |
| No | 9 | 44 | 55 | 0.5-8.1 |  |
| Yes | 30 | 23 | 75 | 0.1-1.9 |  |
| **Progesterone receptor** |  |  |  |  | 0.7 |
| No | 12 | 25 | 72 | 0.2-2.9 |  |
| Yes | 27 | 29 | 69 | 0.3-4.3 |  |
| **Hormonal therapy** |  |  |  |  | 0.3 |
| Yes | 30 | 27 | 72 | 0.1-1.9 |  |
| No | 10 | 40 | 53 | 0.5-8.2 |  |
| **Chemotherapy** |  |  |  |  | 0.2 |
| Yes | 3 | 0 | 100 | 0.04-2.3 |  |
| No | 37 | 12 | 65 | 0.4-21.07 |  |
| **Radiotherapy** |  |  |  |  | **0.003** |
| Yes | 27 | 19 | 81 | 0.03-0.5 |  |
| No | 13 | 54 | 37 | 1.9-28.6 |  |

Results of survival analysis according to Kaplan Meier method and p values from Mantel-Cox (log-rank) test
