# Supplementary material for: Prognostic impact of circulating Her-2-reactive T-cells producing pro- and/or anti-inflammatory cytokines in elderly breast cancer patients
Source: J Immunother Cancer. 2015 Oct 20;3:45. doi: 10.1186/s40425-015-0090-0 (PMC4617728; doi:10.1186/s40425-015-0090-0)
Supplement: Additional file 2: Table S1. — Clinico-pathological characteristics of younger and older patients. (DOCX 15 kb) [file 40425_2015_90_MOESM2_ESM.docx]

**Table S1: Clinico-pathological characteristics**

| Clinico pathological parameters | Young n=35 | Old  n=40 |
| --- | --- | --- |
| Age range (Years) | 28-53 | 65-87 |
| Median (Years) | 47 | 75 |
| **Tumour stage** |  |  |
| 0 | 1 | 3 |
| 1 | 12 | 18 |
| 2 | 13 | 6 |
| 3 | 6 | 6 |
| 4 | 1 | 6 |
| Unknown | 2 | 3 |
| **Tumour size** |  |  |
| Tis | 2 | 2 |
| T0 | 0 | 1 |
| T1 | 18 | 21 |
| T2 | 13 | 8 |
| T3 | 1 | 3 |
| T4 | 0 | 5 |
| Unknown | 1 | 0 |
| **Nodal status** |  |  |
| No | 17 | 24 |
| N1 | 10 | 6 |
| N2 | 3 | 4 |
| N3 | 2 | 2 |
| Unknown | 3 | 4 |
| **Metastasis** |  |  |
| M0 | 32 | 33 |
| M1 | 1 | 6 |
| Unknown | 2 | 1 |
| **Receptor status** |  |  |
| Triple Negative | 5 | 8 |
| Oestrogen receptor+ | 25 | 33 |
| Progesterone receptor+ | 22 | 31 |
| Her-2: 0 | 15 | 20 |
| Her2: 1 | 11 | 9 |
| Her-2: 2+ | 3 | 5 |
| Her2: 3+ | 4 | 4 |
| Her-2: Unknown | 2 | 2 |
