# Supplementary figures and images for: Prognostic impact of circulating Her-2-reactive T-cells producing pro- and/or anti-inflammatory cytokines in elderly breast cancer patients
Source: J Immunother Cancer. 2015 Oct 20;3:45. doi: 10.1186/s40425-015-0090-0 (PMC4617728; doi:10.1186/s40425-015-0090-0)

## Slide 1
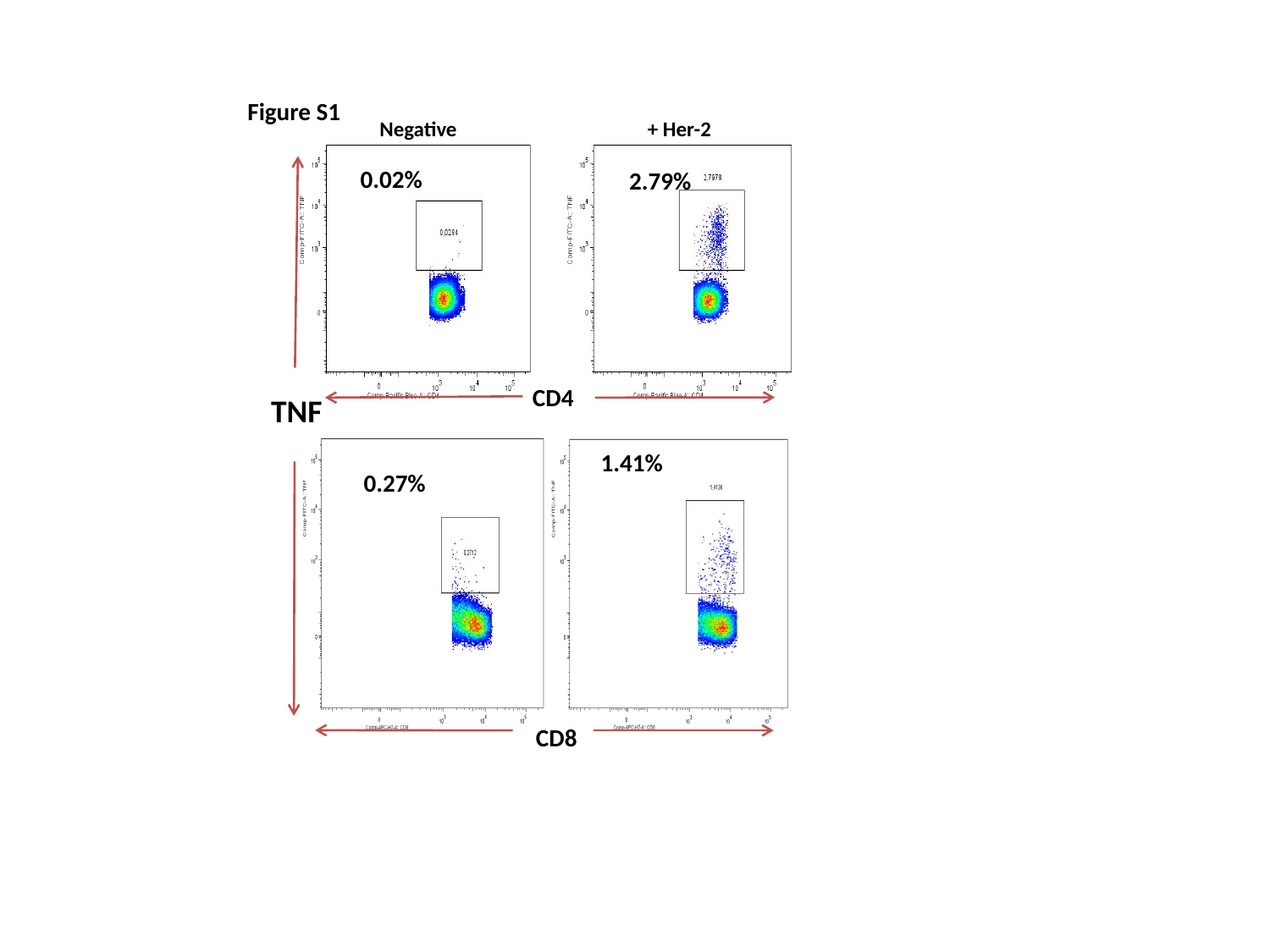

Figure S1
Negative
+ Her-2
0.02%
2.79%
	 CD4
TNF
1.41%
0.27%
	 CD8

Supplement: Additional file 3: Figure S1. — CD8+ and CD4+ T-cell response to Her-2: A representative plot of control and Her-2-stimulated cytokine (eg.TNF-producing CD8+ T-cells and CD4+ T-cells). (PPTX 89 kb) [file 40425_2015_90_MOESM3_ESM.pptx]
